# A Reference Hand-Book of the Medical Sciences

**Published:** 1888-09

**Authors:** 


### Book Reviews.

A Reference Hand-Book of the Medical Sciences, Embracing the Entire Range of Scientific and Practical Medicine and Allied Sciences. By various writers. Illustrated by chromo-lithograph and fine wood engravings. Edited by Albert H. Buck, M. D., New York city. Vol. VI. New York: William Wood & Co., 56 and 58 Lafayette Place. 1888.

Having passed a favorable judgment upon each of the five preceding volumes of this work, as they appeared before the medical public, it is with much satisfaction that, upon a careful examination of the contents of this volume, it is found to come up to the high standard of practical utility observed in the others. It is noted that the number of pages in this, being 778, falls short of the other volumes, which contain respectively in the order of publication, 808, 814, 813, 814 and 813 pages. But the number of contributors being in this 92, exceeds those in some of the preceding, counting up 90, 104, 91, 95 and 86, respectively. There are more names of contributors in this volume, which are not so familiar in medical literature, as those in the previous volumes; but it should be noted that it is not those who write most who write best, and it is one of the chief recommendations of a work of this nature that members of the profession who are known to be well acquainted with a subject are selected as writers on the various topics. It is found that for the most part each has done his part very satisfactorily.

The eminently practical character of this work is shown by enumerating some of the topics of interest to all practitioners, which are presented in this volume as follows: Pregnancy, with ample illustrations; Presbyopia; Prescription-writing; Prostate; Ptomaines; Puerperal Condition; Puerperal Fever; Arterial Pulse; Purpura; Putrefaction; Pylorectomy; Quarantine; Rabies; Railway Medical Service; Railway Spine; Recto-vaginal Fistula; Manual Explorations of the Rectum; Reflexes; Registration of Diseases; Poisonous Reptiles; Resections of Joints, with drawings of instruments and operations; Respiration, with illustrations; Acute and Chronic Articular Rheumatism; Gonorrhœal Rheumatism; Rhinoscopy; Rickets; Roetheln; Sacro-iliac Disease; Salicylic Acid and Salicylates; Sanitary Inspection; Sarcoma; Scapula; Sciatica; Scurvy; Sea-sickness; Secretion; Seminal Incontinence; Septicæmia and Pyæmia; Sewage and Sewerage, with illustrations; Shock; Shoulder Joint; Skin Grafting; Skin Transportation; Skull; Sleep; Small-pox; Spectroscopy; Sphygmograph, with drawings; Spinal Abscess; Spinal Diseases and Injuries, treated elaborately; Spleen; Splints, with drawings; Sponge Grafting; Sterility in Male and Female; Organic Diseases of the Stomach; Suicide; Superfœtation; Suspension Splint, with drawings; Sympathetic Ophthalmitis; Synovitis; Syphilis; Teeth, with full illustrations; Tendon Reflex and Tenotomy.

Other matters of equal importance go to make up one of the most interesting collections of papers to be found in any recent publication of medical literature.

Among the few subjects to which a brief allusion may be made, three papers near the opening of this volume deserve careful consideration from the reader, viz.: Pregnancy, by Theophilus Parvin, M. D.; Puerperal Condition, by James B. Baird, M. D., and Puerperal Fever, by James D. Etheridge, M. D. They are so allied to each other as to meet most of the requirements of those desiring information in this department, and yet the authors may well say, don't view me with a critic's eye, but pass my imperfections by.

For instance, Parvin does not enlighten us in regard to transverse presentations of the fœtus or placenta previa, though he departs from his original proposition by treating of various abnormal matters of less interest. It certainly must strike all the married members of the profession as a new doctrine that "sexual intercourse during pregnancy is utterly unnatural;" and the tolerance, if not entire acceptability, of the conjugal act on the part of the human female after conception is an exception to what holds with inferior animals. This affords a strong support of the marriage relation of one man with one woman, and tells against polygamy.

There are a few sins of omission in Baird's paper which are notable, as the efficacy of camphor in after-pains, the magical effect of stupes of warm vinegar in threatened mammary inflammation, and the almost specific action of spirits of turpentine in puerperal fever, while he gives no caution against vaginal injections of corrosive sublimate.

The otherwise valuable article of Etheridge is marred by a bald assertion that "the only constant symptoms in all forms of puerperal fever are the facial expression and the pulse ;" and yet does not inform us what peculiar indications of the features or circulation characterize this disease, which might guide the inexperienced practitioner in making a diagnosis.

Another group of papers on kindred diseases calls for a passing notice—Acute Articular Rheumatsm, by James E. Graham, M. D.; Chronic Articular Rheumatism, by N. S. Davis, M. D., and Gonorrhœal Rheumatism, by Norman Bridge, M. D. This designation seems to ignore inflammatory rheumatism involving other structures than those about the joints; and the picture thus fails in correctness, as every physician must have had occasion to deal with that most painful rheumatic development of an acute inflammatory nature involving the muscular tissue and fibrous facia in different portions of the body. Confining our attention to the forms of diseases treated of in these papers, there are some measures of prime importance which should be entitled to more consideration from the profession than they have received from the authors. Aqua ammonia, simply as the best representative of the alkaline treatment, calomel as the typical corrective of secretions and the iodide potash as an alterative, are among the most efficacious means of combatting the rheumatic diathesis in these cases.

Still another trio claims our attention in the articles on Skin Grafting, by W. H. Carmalt, M. D.; Skin Transportation, by the same author, and Sponge Grafting, by Middleton Michel, M. D.

These papers are of great practical utility, and would be appropriately supplemented by some notice of transplantation of the egg membrane to supply a covering for denuded surfaces. This has been done successfully by the writer in one instance, after observing the satisfactory results in the practice of others, and especially the favorable report of this process by Dr. Wilson, of Louisville, which appeared in the *Boston Medical and Surgical Journal* in 1884.

The little importance attached to the pedicles in skin transportation, by Carmalt, is, to say the least, misleading, if intended to apply to such cases of plastic surgery as only have marginal attachments for the portions of skin, and in large surfaces where suppuration may occur in spite of all antiseptic precautions. An instance in the writer's experience of each form will suffice for illustration. The dissection from each cheek of flaps for an artificial nose in which one became shriveled and cold, but through the pedicle had its vitality restored. The lifting of a large flap from between the shoulders to cover a granulating surface extending across the nape of the neck, under which suppuration supervened, but through the large attachment to the sound skin the life was kept up until union by granulation occurred.

Our space does not allow further comments.

